# Two‐Sample Bidirectional Mendelian Randomization Study With Causal Association Between Metabolic Syndrome and Cerebral Aneurysm

**DOI:** 10.1002/brb3.70396

**Published:** 2025-03-04

**Authors:** Yu Li, Kai Zhao

**Affiliations:** ^1^ Department of Neurosurgery, Tongji Hospital, Tongji Medical College Huazhong University of Science and Technology Wuhan China

**Keywords:** Cerebral aneurysm, mendelian randomization, metabolic syndrome, nonruptured, subarachnoid hemorrhage

## Abstract

**Background:**

We used a two‐sample mendelian randomization (MR) method to comprehensively investigate the causality of metabolic syndrome (MetS) or its components, including MetS, triglyceride (TG), high‐density lipoprotein cholesterol (HDL‐C), low‐density lipoprotein cholesterol (LDL‐C), fasting blood glucose (FBG), waist circumference (WC), and hypertension (HP), with cerebral aneurysm including nonruptured and ruptured aneurysmal subarachnoid hemorrhage (SAH).

**Methods:**

By leveraging large‐scale genome‐wide association study (GWAS) summary statistics of MetS or its components and cerebral aneurysm (nonruptured and SAH) from European, MR, reverse‐direction MR, and sensitivity analysis were utilized to quantify the genetic correlations and causal relationships. In addition, we adjusted for multiple comparisons using the false discovery rate (FDR) correction.

**Results:**

Two‐sample MR analysis showed that HP was a risk factor for cerebral aneurysm (nonruptured and SAH) with odds ratio (OR) of 58.959 (95% confidence interval [95% CI] = 12.073–287.920, *p *< 0.001, *q* < 0.001), and 32.290 (95% CI = 5.615–185.671, *p *< 0.001, *q *< 0.001), respectively. HDL‐C (OR = 0.836, 95% CI = 0.728–0.960, *p* = 0.011, *q* = 0.039) and FBG (OR = 0.626, 95% CI = 0.426–0.919, *p* = 0.017, *q* = 0.039) were negatively correlated with cerebral aneurysm (nonruptured). The HDL‐C result was inconsistent after adjusting for TG and LDL‐C by multivariable MR analysis. In reverse MR analysis, we found that there was no statistical causal association between cerebral aneurysm (nonruptured) and MetS or its components. Genetic liability to cerebral aneurysm (SAH) was inversely associated with HDL‐C and FBG but was not associated with others, however, sensitivity analysis showed that few instrumental variables made a big difference.

**Conclusions:**

Genetically determined elevated FBG level reduces the risk of cerebral aneurysm (nonruptured). However, hypertension increases the risk of cerebral aneurysm (nonruptured and SAH).

## Graphical abstract

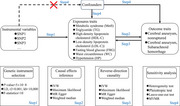

**Abbreviations**
CI: confidence interval
FBG: fasting blood glucose
FDR: false discovery rate
GWAS: genome‐wide association study
HDL‐C: high‐density lipoprotein cholesterol
HP: hypertension
IVs: instrumental variables
IVW: inverse‐variance‐weighted
LDL‐C: low‐density lipoprotein cholesterol
MetS: metabolic syndrome
ML: maximum likelihood
MR: mendelian randomization
MR‐PRESSO: MR pleiotropy residual sum and outlier
MVMR: multivariate MR
OR: odds ratio
SAH: subarachnoid hemorrhage
SNPs: single‐nucleotide polymorphisms
TC: total cholesterol
TG: triglyceride
WC: waist circumference
WM: weighted median

## 1 Background

Metabolic syndrome (MetS) is a complex cluster of metabolic disorders that is usually composed of a group of abnormal conditions, including hyperlipidemia, hyperglycemia, abdominal obesity, and hypertension (HP). There has been a rising trend in the occurrence of MetS, with the affected population increasingly showing a tendency toward younger. It emerges as a pivotal player in the realm of patients and poses a great worldwide threat to the health (Grundy et al. 2004; Váncsa et al. 2023; Zhu and Ding 2023). It has been well documented that MetS alters vascular endothelial in the heart, brain, kidney, and peripheral vessels, and increases the risk of vascular system diseases (Willson et al. 2019). Cerebral aneurysm, nonruptured and ruptured aneurysmal subarachnoid hemorrhage (SAH), is abnormal focal dilation in arteries of intracranial blood vessels, which is not actually tumors within the skull. The situation resembles the formation of a bubble at a vulnerable point in a car tire. At a certain level of pressure, the fragile area in the wall of an intracranial blood vessel could expand and grow, thus forming a latent threat within the brain. Once this aneurysm ruptures, a severe type of stroke, it could lead to subarachnoid hemorrhage, with high rates of mortality and disability (Jin et al. 2022; Li et al. 2021; Liu et al. 2022). Bakker et al. revealed a polygenic architecture and explain over half of the disease heritability, and found that cerebral aneurysm was genetically determined differs considerably among individuals especially in smoking and high blood pressure (Bakker et al. 2020). It has been acknowledged that blood lipids are risk factors for atherosclerosis, however, the role of lipids in the development of cerebral aneurysm remains uncertain (Sabatine et al. 2017; Zhang et al. 2022). More than half of the patients with diabetes mellitus exhibit atherosclerosis, also involved the cerebral arteries (Gu et al. 2006). While studies have suggested that lifestyle, including obesity, could be associated with an increased risk of cardiovascular diseases and aneurysms, and causal association between abdominal obesity and cerebral aneurysm was either insufficient or inconclusive (Liu et al. 2022; Uusitupa et al. 2019).

The associations between MetS or its components including MetS, triglyceride (TG), high‐density lipoprotein cholesterol (HDL‐C), low‐density lipoprotein cholesterol (LDL‐C), fasting blood glucose (FBG), waist circumference (WC), and HP and cerebral aneurysm uncovered through conventional observational studies were prone to be influenced by confounding factors, restricted sample sizes, inadequate follow‐up durations, and potential reverse causality. Mendelian randomization (MR), as a robust approach for causal assessment, is engineered to quantify the direct impact of exposure variables on outcomes. This methodology is particularly potent in mitigating the influence of confounding elements and in addressing the complexities associated with reverse causality, thereby offering a clearer lens through which to view potential causal relationships. In essence, MR leverages genetic variants as instrumental variables (IVs), which are naturally randomized at conception and are therefore less susceptible to environmental confounding. By focusing on these genetic markers that are associated with specific exposures but not directly linked to the outcome except through their effect on the exposure, MR provides a means to approximate the causal effect of an exposure on an outcome without being swayed by typical observational study biases. This strategy, thus, stands as a powerful tool in epidemiological research, enabling investigators to draw stronger inferences about causality than what is typically achievable through traditional observational methods (Cao et al. 2023; Palmer et al. 2011).

To our knowledge, there have been MR studies evaluating the causal relationship between them; one discovered elevated blood pressure significantly increases intracranial aneurysm risk (Zeng et al. 2023) and other demonstrated serum HDL‐C, LDL‐C, total cholesterol (TC), and TG levels dysfunction is a causal risk factor for aneurysms (Y. Chen, Huang, et al. 2021; Zhang et al. 2022). However, MetS encompasses a cluster of metabolic disorders, rather than just being a case of dyslipidemia or high blood pressure. Consequently, employing the technique of MR, we are set to embark on a bidirectional MR into the relationship between MetS or its components and cerebral aneurysm (nonruptured and SAH).

## 2 Methods

### 2.1 Study Design and Data Collection

**FIGURE 1:**
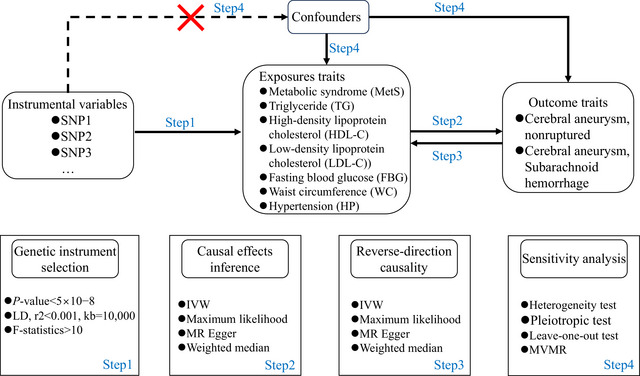
Flow diagram of the study design. IVW, inverse variance weighting; LD, linkage disequilibrium; MR, mendelian randomization; MVMR, multivariate MR.

Figure 1 shows the study design and the assumptions of MR in our study. Using a two‐sample MR analysis with genome‐wide association study (GWAS) summary statistics, we investigated the potential causal effect of genetically predicted MetS or its components on cerebral aneurysm (nonruptured and SAH). We used single‐nucleotide polymorphisms (SNPs) associated with MetS or its components, including MetS dataset (Lind 2019), three datasets (for TG, HDL‐C, and LDL‐C) from UK Biobank (Richardson et al. 2020), fasting blood glucose (FBG) dataset (J. Chen, Spracklen, et al. 2021), waist circumference (WC), and hypertension (HP) dataset (https://gwas.mrcieu.ac.uk/), as IVs for the primary analysis in a GWAS of European ancestry. Summary‐level data for cerebral aneurysm including nonruptured and SAH were obtained from the FinnGen consortium, consisting of 415,119 individuals of European ancestry (3014 cases and 412,105 controls) and 3814 cases and 408,928 controls, respectively (https://www.finngen.fi/en). The detailed information of the seven datasets is shown in Table S1. In each combination of exposure and outcome, samples were obtained from two independent but homogenous populations, both of European ancestry. In the reverse direction MR analysis, we assessed the potential causal effect of genetically predicted cerebral aneurysm (nonruptured and SAH) on MetS or its components. In summary, we performed 28 MR analyses to investigate the bidirectional relationship between cerebral aneurysm (nonruptured and SAH) and MetS. Our study was documented in line with the MR‐STROBE criteria (Skrivankova et al. 2021). Because all data were taken from publicly available summary statistics, ethical approval was not required.

### 2.2 Instrumental Variable Selections

A crucial step of MR was to select appropriate genetic variants to serve as valid IVs for cerebral aneurysm (nonruptured and SAH). Based on the aforementioned above datasets and referring to previous literature (Dong et al. 2021; Zeng et al. 2019), we followed the strict screening procedures. First, we retained variants for MetS or its components with a *p* value smaller than 5 × 10^−8^. Then, we removed highly correlated variants with *r* ^2^ greater than 0.001 in the range of 10 Mb. In addition, we conducted the *F* statistical value (Chen et al. 2022).

$$F=\frac{\left(N-k-1\right)\times R^{2}}{\left(1-R^{2}\right)\times k}$$

$$\;R^{2}=\frac{\beta^{2}}{\beta^{2}+SE^{2}\;\times \;N}\;$$

We ensured that each alternative SNP selected as IV was strongly associated (*F* > 10) with MetS or its components. Conclusively, we only kept independent candidate IVs to inquiry the causal relationship between seven traits and cerebral aneurysm. The details of these IVs are shown in Tables S2–S8. We implemented four two‐sample MR analysis, including fixed‐effects and random‐effects inverse variance weighting (IVW), maximum likelihood (ML), MR Egger, and weighted median (WM) methods, to estimate the prospective causal effect of seven traits on cerebral aneurysm. The IVW analysis constitutes the principal methodology employed in our MR research. Failing to consider the intercept term, the IVW approach regarded the reciprocal of the outcome variance (the square of SE) as the weight. Under the IVW premise, we assume that the IVs are devoid of pleiotropy. Consequently, it is imperative to verify that these IVs do not exhibit pleiotropy when applying the IVW method to avoid biased results (Bowden et al. 2015). To ensure the robustness and reliability of the IVW method, we implemented multiple supporting approaches, including MR‐Egger, WM, and ML methods, which served as essential tools for validating the findings of our study (Bowden et al. 2016; Burgess et al. 2013).

### 2.3 Sensitivity Analysis

We performed a sensitivity analysis to evaluate the suspected violations of the model assumptions following methods: (1) heterogeneity test, (2) pleiotropic test, and (3) leave‐one‐out test. Foremost, we used Cochran's *Q* value which estimated the heterogeneity between IVs to test heterogeneity (Greco et al. 2015), when *p* < 0.05 it was considered to indicate the presence of heterogeneity, and IVW random effect method was conducted as the dominant effect size. Thereafter, the deviation of the intercept from zero in MR Egger regression could be a valid indicator to evaluate the potential existence of horizontal pleiotropy (Burgess and Thompson 2017). Additionally, the MR pleiotropy residual sum and outlier (MR‐PRESSO) method was used to identify horizontal pleiotropy outliers in multi‐instrument summary‐level MR testing and then to reassess causal effects after removing pleiotropy IVs (Verbanck et al. 2018). Eventually, leave‐one‐out analysis was performed by gradually removing an SNP and calculating the causal effect of the remaining SNPs, and observing whether the result significantly changed after removing each SNP (Noyce et al. 2017). Furthermore, we carried out multivariate MR (MVMR) analysis to gain deeper insights into the intricate interplay the independent influence of those on the risk of cerebral aneurysm. Also, we used the false discovery rate (FDR) for multiple analysis, with a significance level of *q* adjusted = 0.05 being the cutoff. Similarly, we also performed reverse‐direction MR to assess the potential reverse causal effects of cerebral aneurysm on MetS or its components. All the analyses are performed by R software (v4.3.0). Specially, we used TwoSampleMR R package (v0.5.7) to perform MR analysis.

## 3 Results

### 3.1 Genetic Instruments

After the SNP with linkage disequilibrium (LD) clumping step, we removed SNPs and palindromic structure, subsequently, a total of 1368 SNPs were identified as IVs for exposure (MetS, FBG, TG, HDL‐C, LDL‐C, WC, and HP), which met the generally accepted genome‐wide significance threshold (*p *< 5 × 10^−8^, *r* ^2 ^< 0.001, kb = 10,000) (Tables S2–S8). In this study, the *F*‐statistics of each SNP we used were greater than the empirical threshold 10, and no bias was found for weak IVs. Following the elimination of SNPs that exhibited outlier via the MR‐PRESSO outlier detection method and MR‐Egger regression analysis, we guaranteed that the residual IVs were uncontaminated by horizontal pleiotropy.

### 3.2 Causal Effects of MetS or Its Components and Cerebral Aneurysm (Nonruptured)

**FIGURE 2:**
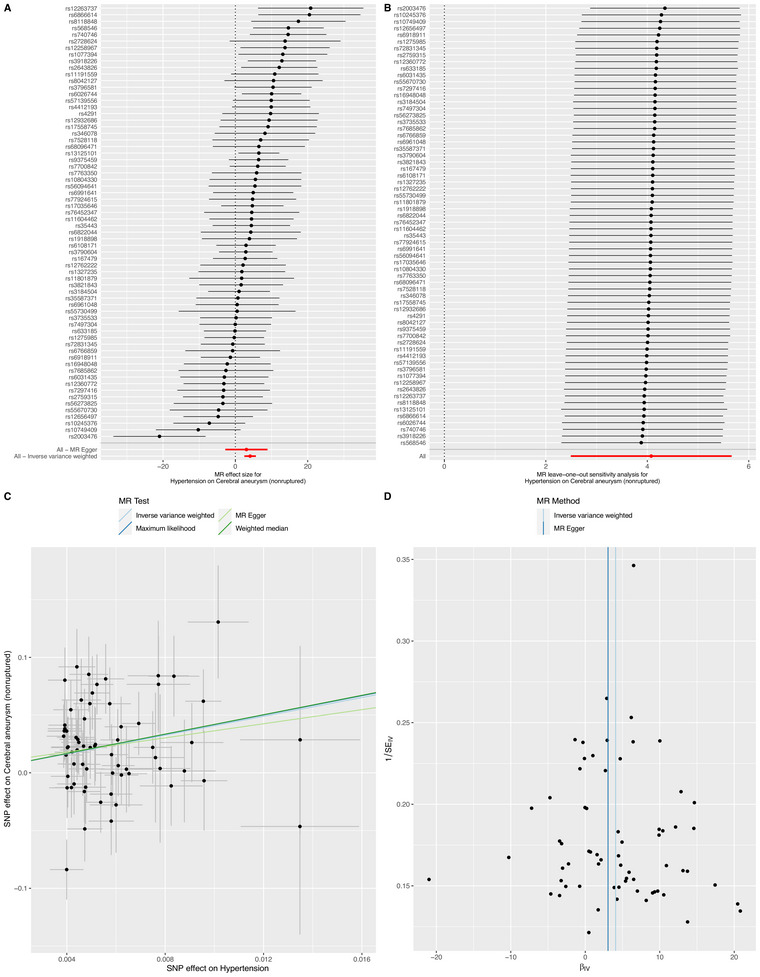
Summary of the MR analysis for hypertension on cerebral aneurysm (nonruptured). (A) MR effect size of each IVs, MR‐Egger, and IVW. (B) Leave‐one‐out sensitivity analysis for hypertension on cerebral aneurysm (nonruptured). (C) The scatter plot of causal effects of hypertension on cerebral aneurysm (nonruptured). We use vertical and horizontal lines to show 95% CI of the estimated effect of IVs on hypertension (x‐axis) and that on cerebral aneurysm (nonruptured) (y‐axis), respectively. (D) The funnel plot of the causal effect of hypertension on cerebral aneurysm (nonruptured). Each point represents the estimated causal effect of each IVs. The vertical dark blue line represents the causal effect estimate obtained using the MR‐Egger method; the light blue line represents the causal effect estimate obtained using the IVW method. IVs, instrumental variables; IVW, inverse variance weighting; MR, Mendelian randomization.

The results of IVW (odds ratio [OR] = 58.959, 95% CI = 12.073–287.920, *p* < 0.001), ML (OR = 65.065, 95% CI = 16.938–249.930, *p* < 0.001), and WM analysis (OR = 66.522, 95% CI = 9.751–453.798, *p* < 0.001) showed that HP increased the risk of cerebral aneurysm (nonruptured). In addition, there were still significant differences after FDR correction (Figure 2A,C and Table 1). The leave‐one‐out method (Figure 2B) and funnel plots (Figure 2D) also reveal the stability of the results of our MR analysis. The MR‐Egger (*p* = 0.008), ML (*p* = 0.012), and IVW (*p* = 0.010) *p* values of the Cochran *Q*‐test indicated significant heterogeneity in our results, and no significant MR‐Egger intercept values (intercept = 0.016; *p* = 0.722) were observed.

**TABLE 1:** MR results of causal association between MetS or its components and cerebral aneurysm (nonruptured).

s	Outcome	Methods	OR (95%)	*p* value	*q* value	Egger intercept	*p* value	Cochran's *Q* test	*p* value
Metabolic syndrome	Cerebral aneurysm (nonruptured)					−0.001	0.891		
		Inverse variance weighted	1.068 (0.959–1.189)	0.231	0.269			87.416	0.242
		Maximum likelihood	1.069 (0.964–1.184)	0.206	0.241			87.394	0.243
		MR Egger	1.084 (0.857–1.369)	0.504	0.587			87.395	0.219
		Weighted median	1.097 (0.943–1.277)	0.231	0.405				
Triglyceride						−0.002	0.568		
		Inverse variance weighted	1.091 (0.953–1.250)	0.205	0.269			338.853	0.008
		Maximum likelihood	1.092 (0.965–1.236)	0.161	0.226			338.843	0.008
		MR Egger	1.139 (0.932–1.392)	0.203	0.485			338.456	0.008
		Weighted median	1.072 (0.865–1.328)	0.526	0.613				
High‐density lipoprotein cholesterol						−0.002	0.609		
		Inverse variance weighted	0.836 (0.728–0.960)	0.011	0.039			402.447	0.001
		Maximum likelihood	0.835 (0.738–0.945)	0.004	0.011			402.400	0.001
		MR Egger	0.872 (0.705–1.079)	0.208	0.485			402.115	0.001
		Weighted median	0.929 (0.753–1.145)	0.488	0.613				
Low‐density lipoprotein cholesterol						−0.007	0.132		
		Inverse variance weighted	0.847 (0.707–1.016)	0.073	0.128			234.970	<0.001
		Maximum likelihood	0.847 (0.732–0.980)	0.025	0.045			234.935	<0.001
		MR Egger	0.982 (0.755–1.276)	0.889	0.890			231.424	<0.001
		Weighted median	0.852 (0.678–1.071)	0.171	0.403				
Fasting blood glucose						0.001	0.904		
		Inverse variance weighted	0.626 (0.426–0.919)	0.017	0.039			85.455	0.021
		Maximum likelihood	0.623 (0.450–0.864)	0.005	0.011			85.478	0.021
		MR Egger	0.604 (0.301–1.210)	0.160	0.485			85.434	0.017
		Weighted median	0.719 (0.447–1.155)	0.173	0.403				
Waist circumference						0.003	0.538		
		Inverse variance weighted	0.958 (0.772–1.189)	0.697	0.697			410.655	0.017
		Maximum likelihood	0.957 (0.782–1.172)	0.673	0.673			410.653	0.017
		MR Egger	0.799 (0.431–1.481)	0.476	0.587			410.211	0.016
		Weighted median	0.993 (0.715–1.380)	0.968	0.968				
Hypertension						0.016	0.722		
		Inverse variance weighted	58.959 (12.073–287.920)	<0.001	<0.001			94.338	0.010
		Maximum likelihood	65.065 (16.938–249.930)	<0.001	<0.001			93.318	0.012
		MR Egger	21.236 (0.063–7131.368)	0.307	0.537			94.150	0.008
		Weighted median	66.522 (9.751–453.798)	<0.001	<0.001				

Under the FDR correction, the MR analysis using the IVW method revealed a significant causal relationship between HDL‐C levels and the risk of cerebral aneurysm (nonruptured) (per SD increase OR = 0.836, 95% CI = 0.728–0.960, *p* = 0.011, *q* = 0.039). Similarly, risk estimation results in ML (OR = 0.835, *p* = 0.004, *q* = 0.011), MR‐Egger (OR = 0.872, *p* = 0.208, *q* = 0.485), and WM (OR = 0.929, *p* = 0.488, *q* = 0.613) methods demonstrate a similar trend although the associations did not reach statistical significance (Table 1 and Figure S1). Notably, there were no indications of horizontal pleiotropy (*p* = 0.609). While, the *p* values obtained from the Cochran *Q*‐test for MR‐Egger, ML, and IVW were both less than 0.05, indicating substantially heterogeneity in the results. Furthermore, to investigate the independent influence of TG, HDL‐C, and LDL‐C on the risk of cerebral aneurysm (nonruptured), given their close correlation, we employed MVMR analysis. Ultimately, the results of the MVMR analysis, with adjustments made for HDL‐C using the IVW method, demonstrated no direct causal effect of HDL‐C levels on the risk of cerebral aneurysm (OR = 0.911, 95% CI = 0.754–1.100, *p* = 0.331). Instrumental strength was found for HDL‐C (conditional *F* = 52.766), LDL cholesterol (conditional *F* = 45.549), and triglycerides (conditional *F* = 41.902).

Under the FDR correction, the IVW method revealed a significant causal relationship between FBG and the cerebral aneurysm (nonruptured) (OR = 0.626, 95% CI = 0.426–0.919, *p* = 0.017, *q* = 0.039). Similarly, risk estimation results in ML (OR = 0.623, *p* = 0.005, *q* = 0.011), MR‐Egger (OR = 0.604, *p* = 0.160, *q* = 0.485), and WM (OR = 0.719, *p* = 0.173, *q* = 0.403) methods demonstrate a similar trend although no statistical significance (Table 1). Notably, there were no indications of horizontal pleiotropy (*p* = 0.904). While, the *p* values obtained from the Cochran *Q*‐test for MR‐Egger (Cochrane's *Q* = 249.668, *p* < 0.001), ML (Cochrane's *Q* = 249.891, *p* < 0.001), and IVW (Cochrane's *Q* = 249.952, *p *< 0.001) were both less than 0.05, indicating substantially heterogeneity in the results. The forest plot illustrated that genetically predicted FBG was significantly associated with cerebral aneurysm (nonruptured) (Figure S2).

However, no significant associations of favorable or unfavorable remaining four components (MetS, TG, LDL‐C, and WC level) with cerebral aneurysm (nonruptured) were detected (Table 1).

### 3.3 Causal Effects of MetS or Its Components and Cerebral Aneurysm (Subarachnoid Hemorrhage)

**FIGURE 3:**
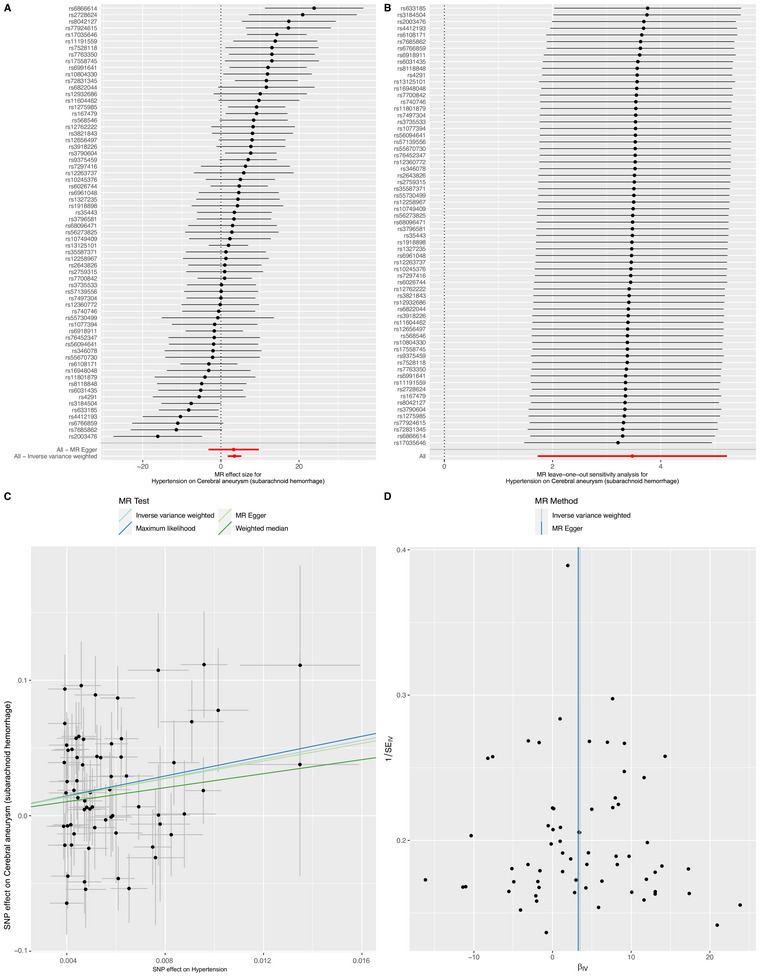
Summary of the MR analysis for hypertension on cerebral aneurysm (subarachnoid hemorrhage). (A) MR effect size of each IVs, MR‐Egger, and IVW. (B) Leave‐one‐out sensitivity analysis for hypertension on cerebral aneurysm (subarachnoid hemorrhage). (C) The scatter plot of causal effects of hypertension on cerebral aneurysm (subarachnoid hemorrhage). We use vertical and horizontal lines to show 95% CI of the estimated effect of IVs on hypertension (x‐axis) and that on cerebral aneurysm (subarachnoid hemorrhage) (y‐axis), respectively. (D) The funnel plot of the causal effect of hypertension on cerebral aneurysm (subarachnoid hemorrhage). Each point represents the estimated causal effect of each IVs. The vertical dark blue line represents the causal effect estimate obtained using the MR‐Egger method; the light blue line represents the causal effect estimate obtained using the IVW method. IVs, instrumental variables; IVW, inverse variance weighting; MR, Mendelian randomization.

Using the IVW method, HP was associated with increased SAH risk (OR = 32.290, 95% CI = 5.615–185.671, *p* < 0.001) (Figure 3 and Table 2). ML, MR Egger, and WM methods consistently demonstrated a causal association between HP and SAH, therefore, strengthening the robustness of our primary findings. Furthermore, after FDR correction, only IVW method of the above results remained statistically significant (*q* < 0.001). Additional detailed information could be found in Figure 3, as well as Table 2 providing further insights into the data. However, there was no statistical causal association with MetS, TG, HDL‐C, LDL‐C, FBG, and WC after FDR correction.

**TABLE 2:** MR results of causal association between MetS or its components and cerebral aneurysm (subarachnoid hemorrhage).

Exposure	Outcome	Methods	OR (95%)	*p* value	*q* value	Egger intercept	*p* value	Cochran's *Q* test	*p* value
Cerebral aneurysm (subarachnoid hemorrhage)	Metabolic syndrome					0.007	0.984		
		Inverse variance weighted	1.035 (0.942–1.136)	0.477	0.557			84.311	0.321
		Maximum likelihood	1.035 (0.945–1.134)	0.460	0.518			84.308	0.321
		MR Egger	1.033 (0.842–1.266)	0.758	0.885			84.311	0.293
		Weighted median	1.103 (0.955–1.274)	0.182	0.588				
	Triglyceride					−0.002	0.493		
		Inverse variance weighted	1.037 (0.913–1.177)	0.577	0.577			373.239	0.000
		Maximum likelihood	1.037 (0.929–1.157)	0.518	0.518			373.237	0.000
		MR Egger	1.088 (0.902–1.312)	0.378	0.736			372.609	0.000
		Weighted median	1.093 (0.912–1.309)	0.336	0.588				
	High‐density lipoprotein cholesterol					0.003	0.313		
		Inverse variance weighted	0.881 (0.787–0.987)	0.029	0.100			339.179	0.198
		Maximum likelihood	0.881 (0.789–0.983)	0.023	0.082			339.157	0.198
		MR Egger	0.823 (0.692–0.980)	0.029	0.203			338.089	0.199
		Weighted median	0.899 (0.737–1.097)	0.295	0.588				
	Low‐density lipoprotein cholesterol					0.005	0.185		
		Inverse variance weighted	0.888 (0.747–1.056)	0.179	0.417			271.177	<0.001
		Maximum likelihood	0.889 (0.781–1.012)	0.076	0.177			271.154	<0.001
		MR Egger	1.005 (0.782–1.292)	0.969	0.969			268.003	<0.001
		Weighted median	0.929 (0.747–1.154)	0.504	0.588				
	Fasting blood glucose					0.007	0.991		
		Inverse variance weighted	0.840 (0.601–1.174)	0.307	0.430			81.780	0.039
		Maximum likelihood	0.839 (0.627–1.122)	0.235	0.337			81.767	0.039
		MR Egger	0.843 (0.460–1.545)	0.582	0.814			81.780	0.032
		Weighted median	0.908 (0.576–1.431)	0.678	0.678				
	Waist circumference					0.002	0.649		
		Inverse variance weighted	0.900 (0.742–1.089)	0.277	0.430			409.387	0.019
		Maximum likelihood	0.898 (0.750–1.075)	0.241	0.337			409.373	0.019
		MR Egger	0.798 (0.460–1.382)	0.421	0.736			409.146	0.017
		Weighted median	0.877 (0.637–1.209)	0.424	0.588				
	Hypertension					0.001	0.947		
		Inverse variance weighted	32.290 (5.615–185.671)	<0.001	<0.001			144.225	<0.001
		Maximum likelihood	65.065 (11.666–130.784)	<0.001	<0.001			142.768	<0.001
		MR Egger	26.171 (0.042–16390.113)	0.324	0.736			144.215	<0.001
		Weighted median	13.288 (1.738–101.607)	0.013	0.089				

### 3.4 Reverse‐Direction Mendelian Randomization Analysis

In order to identify potential confounding factors that mislead the direction of causal effects, we performed reverse‐direction MR. When cerebral aneurysm (nonruptured) was considered as the exposure, we found that there was no statistical causal association with MetS or its components included MetS, TG, HDL‐C, LDL‐C, WC, and HP using IVW method estimation (Table S9). Although genetic liability to cerebral aneurysm (SAH) was inversely associated with HDL‐C and FBG but was not associated with MetS, TG, LDL‐C, WC, and HP (Table S10), sensitivity analysis showed that few IVs made a big difference (Figures S3 and S4).

## 4 Discussion

To investigate the causal effects of MetS or its components on cerebral aneurysm (nonruptured and SAH), we leveraged summary statistics from large GWAS of European ancestry to perform two‐sample MR and reverse‐direction MR analysis. Our MR results confirmed a direct causal relationship between HP and the risk of cerebral aneurysm (nonruptured and SAH). Moreover, the MR analysis showed that increased HDL‐C and FBG levels were significantly associated with decreased cerebral aneurysm (nonruptured) risk. There was no strong evidence of a causal inference between any other components (MetS, TG, LDL‐C, and WC) and cerebral aneurysm (nonruptured and SAH).

HP is a common comorbid condition in patients with cerebral aneurysm, which can lead to the formation of new aneurysms and the enlargement of pre‐existing ones (Xin et al. 2020; Zhong et al. 2022). Our IVW method verified that HP is a risk for cerebral aneurysm (nonruptured and SAH). Additional sensitivity analysis showed no indication of horizontal pleiotropy interfering with the causal inference, confirmed by the MR‐Egger regression (*p* = 0.722, *p* = 0.947). Ali et al. have shown that compared with normal blood pressure, HP increases the expression of tumor necrosis factor, which promotes the expression of inflammatory cytokines and matrix metalloproteinase, leading to the formation of cerebral aneurysm (Ali et al. 2013). High blood pressure could lead to the formation of blood flow eddies due to the generation of high wall shear stress. The prolonged and intense impact force of the blood flow can continuously act on the cerebral blood vessel walls, leading to abnormal cerebral hemodynamics, cerebral basal arterial network variations, and damage of the vascular endothelial cells. This in turn triggers and amplifies inflammatory responses, causing the destruction and increased susceptibility to necrosis of vascular smooth muscle cells. Furthermore, it results in localized thinning of the cerebrovascular walls, thereby fostering the formation and progression of cerebral aneurysm (Signorelli, Sela, et al. 2018). Moreover, Young's modulus tests showed that alteration of aneurysm wall biomechanics was associated with the formation and rupture of cerebral aneurysm (Signorelli, Pailler‐Mattei, et al. 2018).

Cholesterol is a vital component of cellular membranes and plays a crucial role in the functioning of cells (Segatto et al. 2014). Lipid levels in the blood are acknowledged as contributors to the development of atherosclerosis, and therapies aimed at regulating these lipids have proven effective in decreasing the likelihood of atherosclerotic‐related diseases (Zhang et al. 2022). However, the role of lipids, especially in HDL‐C, in cerebral aneurysm remained unclear. Furthermore, HDL‐C possesses multiple beneficial properties including anti‐inflammatory, anti‐apoptotic, antioxidative, and antithrombotic effects (Pownall and Gotto 2016). These protective functionalities of HDL‐C could account for its role in lowering the risk. This IVW analysis revealed that elevated HDL‐C level did not decrease the risk of cerebral aneurysm (SAH), however, decreased the risk of cerebral aneurysm (nonruptured). Notably, this association disappeared after adjustments through MVMR for potential confounding variables such as TG and LDL‐C. Conclusively, we did not find evidence of a causal relationship between genetically HDL‐C level, and cerebral aneurysm. Our findings suggest that genetically elevated FBG level was related to a low cerebral aneurysm (nonruptured) risk. The underlying pathophysiological mechanisms of FBG in the development of intracranial aneurysm were still unclear. It has been hypothesized that low FBG levels could lead to a more fragile cerebrovascular endothelium ultimately inducing intracranial aneurysm. As far as we known, direct studies specifically investigating the relationship between FBG and cerebral aneurysms might be lacking, previous studies indicated that hyperglycemia could lead to endothelial dysfunction, exacerbate inflammatory responses, induce changes in cerebral vessel walls, and accelerate the process of atherosclerosis (de la Monte et al. 1985; Wang et al. 2021). Therefore, future investigations requiring larger datasets would be necessary to more thoroughly examine the nature of this association.

In our study, we found no evidence for a genetic causal association between genetic variants associated with MetS, TG, LDL‐C, and WC and risk of cerebral aneurysm (nonruptured and SAH). MetS is a complex disease state characterized by abdominal obesity, high blood pressure, high TG, and LDL‐C level. Together, these factors increase the risk of cardiovascular disease. High TG and LDL‐C level might increase the risk of atherosclerosis, which could lead to narrowing of intracranial arteries or indirectly affect the stability of aneurysms (Wang et al. 2021). Individuals with MetS frequently have other cardiovascular risk factors like smoking, obesity, and lack of physical activity, which could also indirectly affect the development and progression of cerebral aneurysm (Bakker et al. 2020; S. Chen, Mao, et al. 2021; Etminan et al. 2020). The investigation into the precise connection between MetS and cerebral aneurysm remains relatively scarce. Moreover, reverse‐direction MR showed that genetic liability to cerebral aneurysm (SAH) was inversely associated with HDL‐C and FBG, however, sensitivity analysis showed that few IVs made a big difference. Further studies are required to elucidate the causal link between these two conditions and to understand how managing MetS could potentially lower the risk of developing cerebral aneurysm.

To our best knowledge, the strength of our MR analysis is that this is the first comprehensive MR study to report the causal association of MetS or its components on the risk of cerebral aneurysm (nonruptured and SAH). However, there are several limitations of this study. To begin with, we discovered that horizontal pleiotropy was not significant in the MR Egger regression analysis; however, as with all MR studies, we could not address unobserved pleiotropy. In addition, according to the International Diabetes Federation (IDF), MetS is a cluster of conditions that are not a single characteristic level (Liu et al. 2006). Then, previous studies have found that smoking and high blood pressure were the strongest risk factors for cerebral aneurysm. In our MVMR analysis, we have not taken those into consideration as covariates. As the final point, the causal effect could not be generalizable to overall populations because of included summary data from GWAS of only European descent. Furthermore, the lack of raw individual‐level and those like comorbid conditions or other environmental factors beyond those addressed data restricted our ability to conduct further analysis on causal effects. Aneurysms could arise at any age due to variations in the cerebral basal arterial network. Going forward, the early identification of these variations in infants is essential to explore the possible associations between metabolic syndrome and aneurysm development in younger individuals.

## 5 Conclusion

In conclusion, leveraging summary data from GWAS of European ancestry, two‐sample MR analysis indicates that elevated FBG level reduces the risk of cerebral aneurysm (nonruptured). However, HP increases the risk of cerebral aneurysm (nonruptured and SAH).

## Author Contributions

**Yu Li**: formal analysis, data curation, Methodology, software, validation, visualization, writing – original draft. **Kai Zhao**: conceptualization, resources, project administration, investigation, supervision, writing – review and editing.

## Ethics Statement

All data were taken from publicly available summary statistics, ethical approval was not required.

## Conflicts of Interest

The authors declare no conflicts of interest.

### Peer Review

The peer review history for this article is available at https://publons.com/publon/10.1002/brb3.70396.

## Supporting information



Supporting Information

Supporting Information

Supporting Information

Supporting Information

Supporting Information

Supporting Information

## Data Availability

The data that support the findings of this study are openly available in FinnGen consortium at https://www.finngen.fi/en.
