# Supplementary material for: Two‐Sample Bidirectional Mendelian Randomization Study With Causal Association Between Metabolic Syndrome and Cerebral Aneurysm
Source: Brain Behav. 2025 Mar 4;15(3):e70396. doi: 10.1002/brb3.70396 (PMC11879889; doi:10.1002/brb3.70396)
Supplement: Supplementary file 1 — Supporting Information [file BRB3-15-e70396-s003.docx]

**A** **two-sample bidirectional** **mendelian randomization study with causal association between** **metabolic syndrome and** **cerebral aneurysm**

Yu Li^1^, Kai Zhao^1*^

1 Department of Neurosurgery, Tongji Hospital, Tongji Medical College, Huazhong University of Science and Technology, Wuhan, 430030, PR China.

*Corresponding Author: Kai Zhao, E-mail: zhaokai@tjh.tjmu.edu.cn

This file includes legends for **Supplemental Tables S1-10** and **Supplemental Figures S1-4**.

**Supplemental Table S1.** Information of GWAS summary statistics.

**Supplemental Table S2.** Instrumental SNPs from metabolic syndrome GWAS.

**Supplemental Table S3.** Instrumental SNPs from triglycerides GWAS.

**Supplemental Table S4.** Instrumental SNPs from HDL cholesterol GWAS.

**Supplemental Table S5.** Instrumental SNPs from LDL cholesterol GWAS.

**Supplemental Table S6.** Instrumental SNPs from fasting blood glucose cholesterol GWAS.

**Supplemental Table S7.** Instrumental SNPs from waist circumference GWAS.

**Supplemental Table S8.** Instrumental SNPs from hypertension GWAS.

**Supplemental Table S9.** Reverse-direction MR results of causal association between Mets or its components and cerebral aneurysm (nonruptured).

**Supplemental Table S10.** Reverse-direction MR results of causal association between Mets or its components and cerebral aneurysm (subarachnoid hemorrhage).

**Supplemental** **Figure S1.** Summary of the MR analysis for HDL-C on cerebral aneurysm (nonruptured). (A) MR effect size of each IVs, MR-Egger, and IVW. (B) Leave-one-out sensitivity analysis for HDL-C on cerebral aneurysm (nonruptured). (C) The scatter plot of causal effects of HDL-C on cerebral aneurysm (nonruptured). We use vertical and horizontal lines to show 95% CI of the estimated effect of IVs on HDL-C (x-axis) and that on cerebral aneurysm (nonruptured) (y-axis), respectively. (D) The funnel plot of the causal effect of HDL-C on cerebral aneurysm (nonruptured). Each point represents the estimated causal effect of each IVs. The vertical dark blue line represents the causal effect estimate obtained using the MR-Egger method; the light blue line represents the causal effect estimate obtained using the IVW method. MR, Mendelian randomization; HDL-C, high-density lipoprotein cholesterol; IVs, instrumental variables; IVW, inverse variance weighting.

**Supplemental** **Figure S2.** Summary of the MR analysis for FBG on cerebral aneurysm (nonruptured). (A) MR effect size of each IVs, MR-Egger, and IVW. (B) Leave-one-out sensitivity analysis for FBG on cerebral aneurysm (nonruptured). (C) The scatter plot of causal effects of FBG on cerebral aneurysm (nonruptured). We use vertical and horizontal lines to show 95% CI of the estimated effect of IVs on FBG (x-axis) and that on cerebral aneurysm (nonruptured) (y-axis), respectively. (D) The funnel plot of the causal effect of FBG on cerebral aneurysm (nonruptured). Each point represents the estimated causal effect of each IVs. The vertical dark blue line represents the causal effect estimate obtained using the MR-Egger method; the light blue line represents the causal effect estimate obtained using the IVW method. MR, Mendelian randomization; FBG, fasting blood glucose; IVs, instrumental variables; IVW, inverse variance weighting.

**Supplemental** **Figure S3**. Summary of the MR analysis for HDL-C on cerebral aneurysm (subarachnoid hemorrhage). (A) MR effect size of each IVs, MR-Egger, and IVW. (B) Leave-one-out sensitivity analysis for HDL-C on cerebral aneurysm (subarachnoid hemorrhage). (C) The scatter plot of causal effects of HDL-C on cerebral aneurysm (subarachnoid hemorrhage). We use vertical and horizontal lines to show 95% CI of the estimated effect of IVs on HDL-C (x-axis) and that on cerebral aneurysm (subarachnoid hemorrhage) (y-axis), respectively. (D) The funnel plot of the causal effect of HDL-C on cerebral aneurysm (subarachnoid hemorrhage). Each point represents the estimated causal effect of each IVs. The vertical dark blue line represents the causal effect estimate obtained using the MR-Egger method; the light blue line represents the causal effect estimate obtained using the IVW method. MR, Mendelian randomization; HDL-C, high-density lipoprotein cholesterol; IVs, instrumental variables; IVW, inverse variance weighting.

**Supplemental** **Figure S4**. Summary of the MR analysis for FBG on cerebral aneurysm (subarachnoid hemorrhage). (A) MR effect size of each IVs, MR-Egger, and IVW. (B) Leave-one-out sensitivity analysis for FBG on cerebral aneurysm (subarachnoid hemorrhage). (C) The scatter plot of causal effects of FBG on cerebral aneurysm (subarachnoid hemorrhage). We use vertical and horizontal lines to show 95% CI of the estimated effect of IVs on FBG (x-axis) and that on cerebral aneurysm (subarachnoid hemorrhage) (y-axis), respectively. (D) The funnel plot of the causal effect of FBG on cerebral aneurysm (subarachnoid hemorrhage). Each point represents the estimated causal effect of each IVs. The vertical dark blue line represents the causal effect estimate obtained using the MR-Egger method; the light blue line represents the causal effect estimate obtained using the IVW method. MR, Mendelian randomization; FBG, fasting blood glucose; IVs, instrumental variables; IVW, inverse variance weighting.
