# Supplementary figures and images for: Two‐Sample Bidirectional Mendelian Randomization Study With Causal Association Between Metabolic Syndrome and Cerebral Aneurysm
Source: Brain Behav. 2025 Mar 4;15(3):e70396. doi: 10.1002/brb3.70396 (PMC11879889; doi:10.1002/brb3.70396)

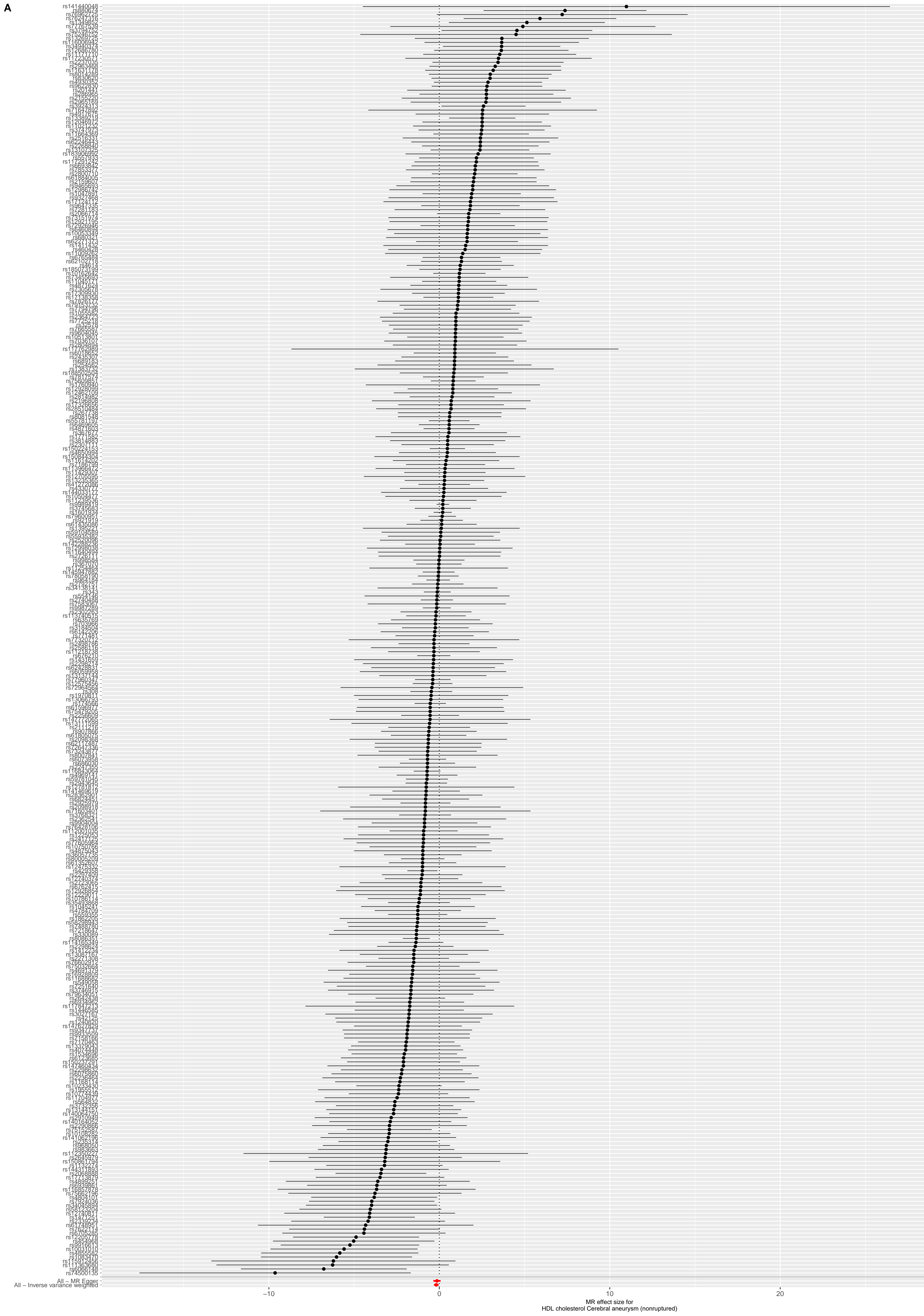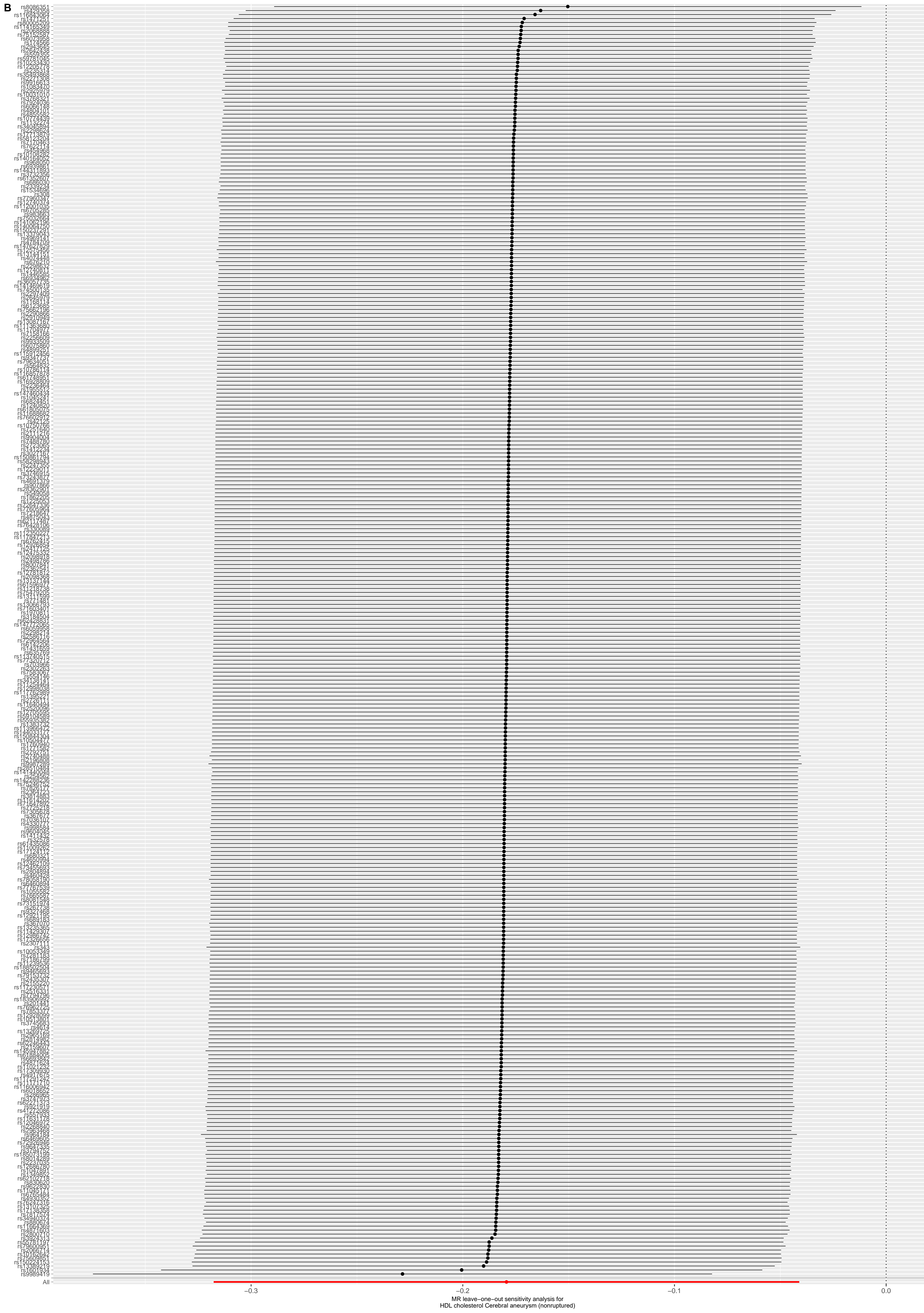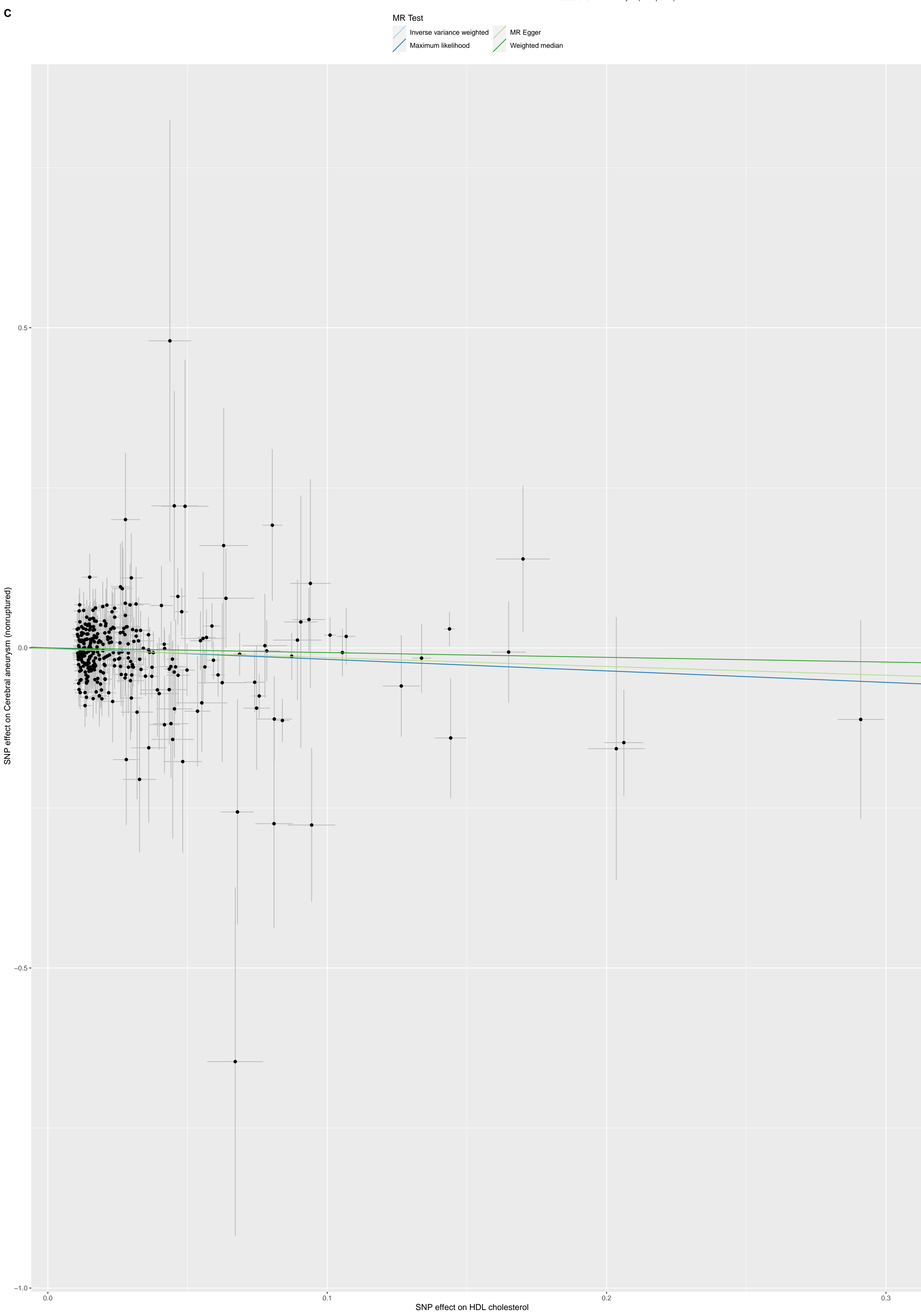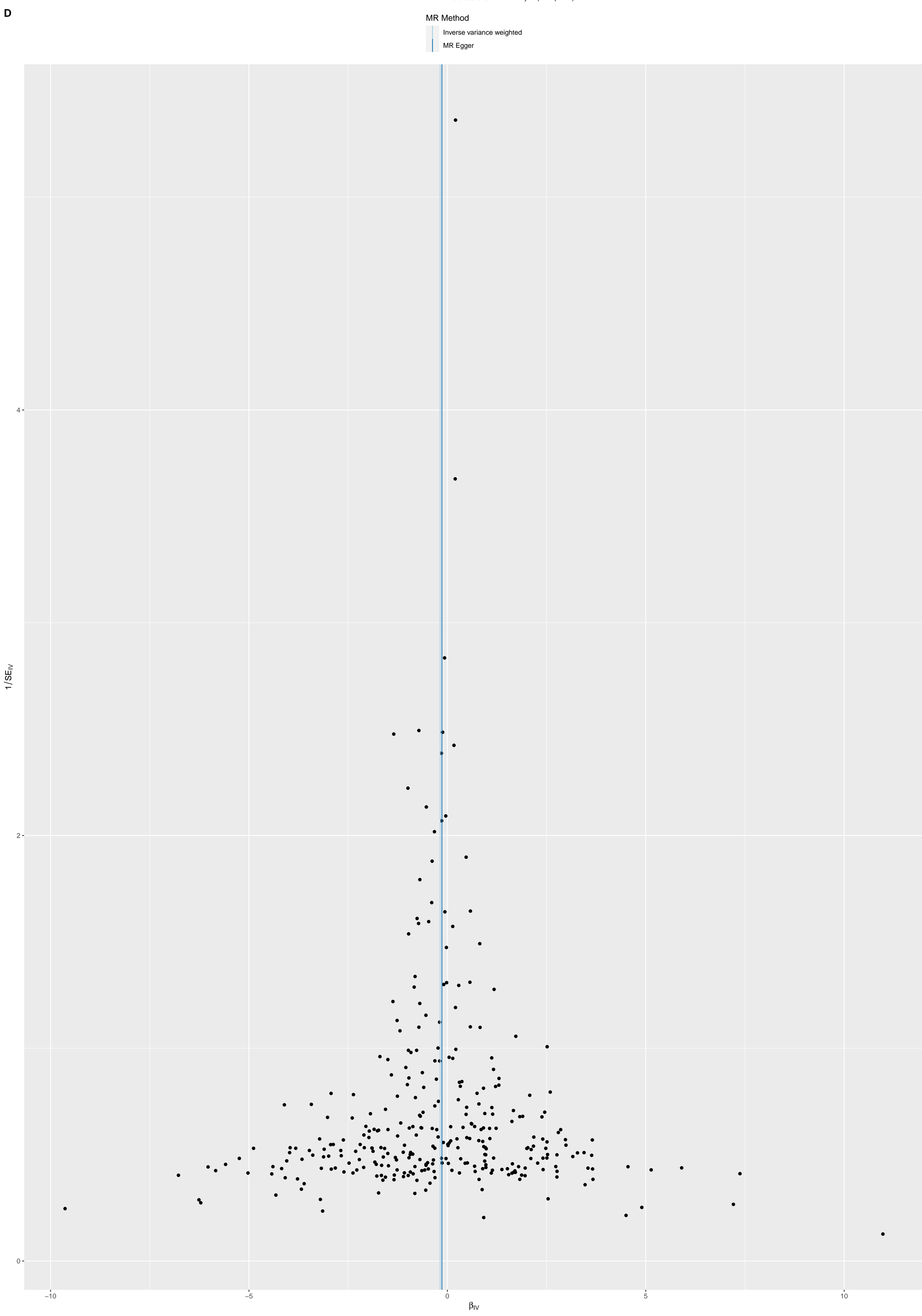

Supplement: Supplementary file 2 — Supporting Information [file BRB3-15-e70396-s006.pdf]

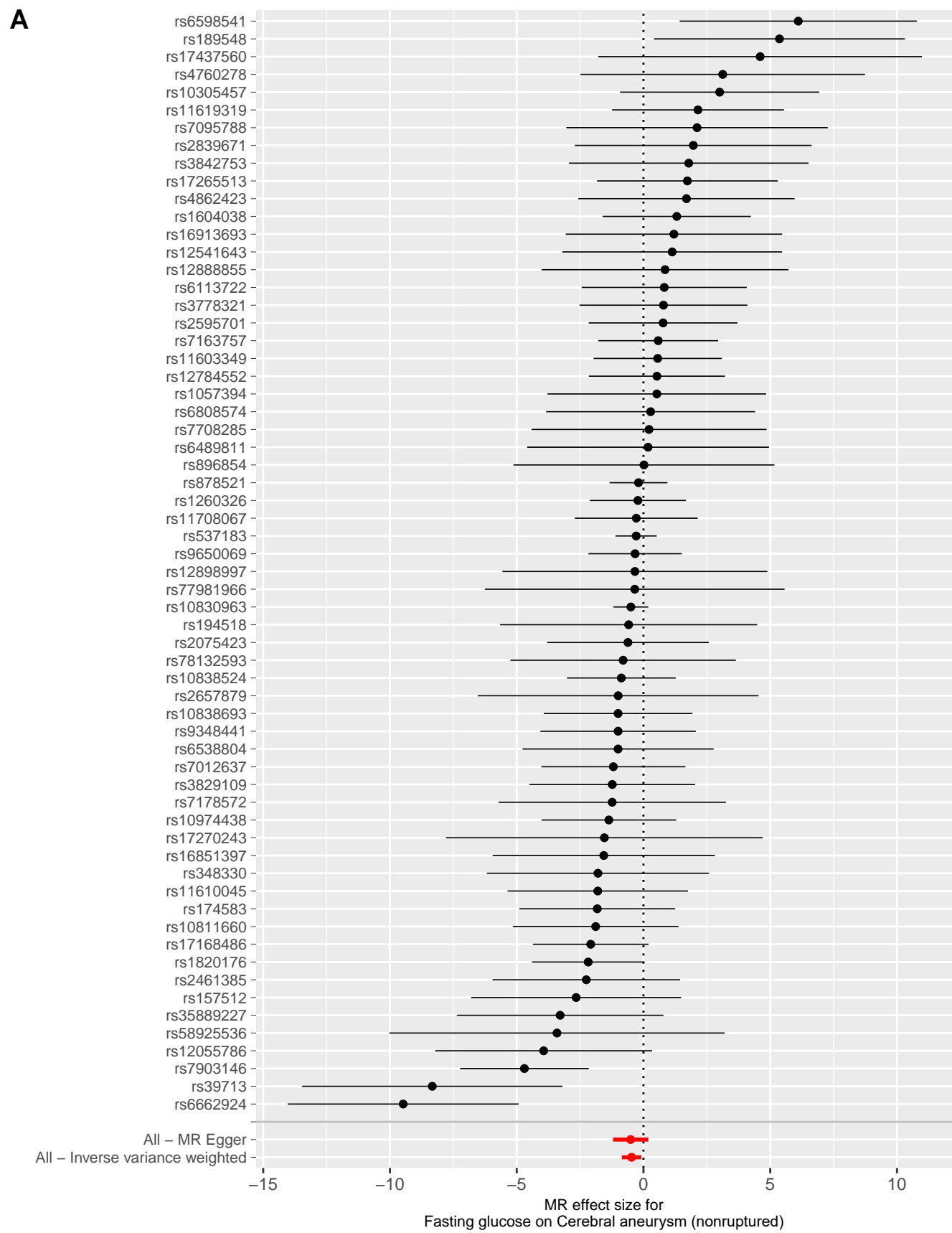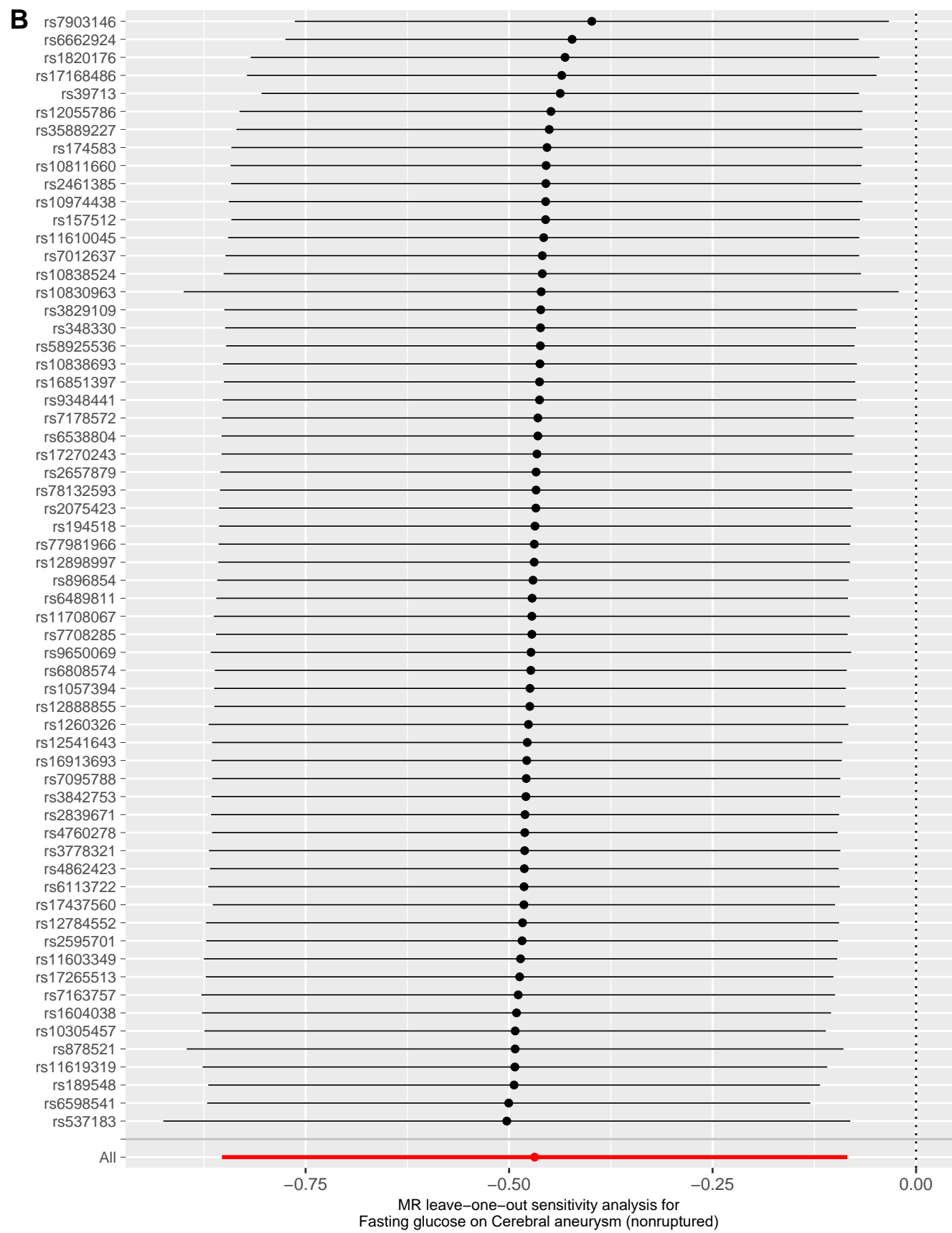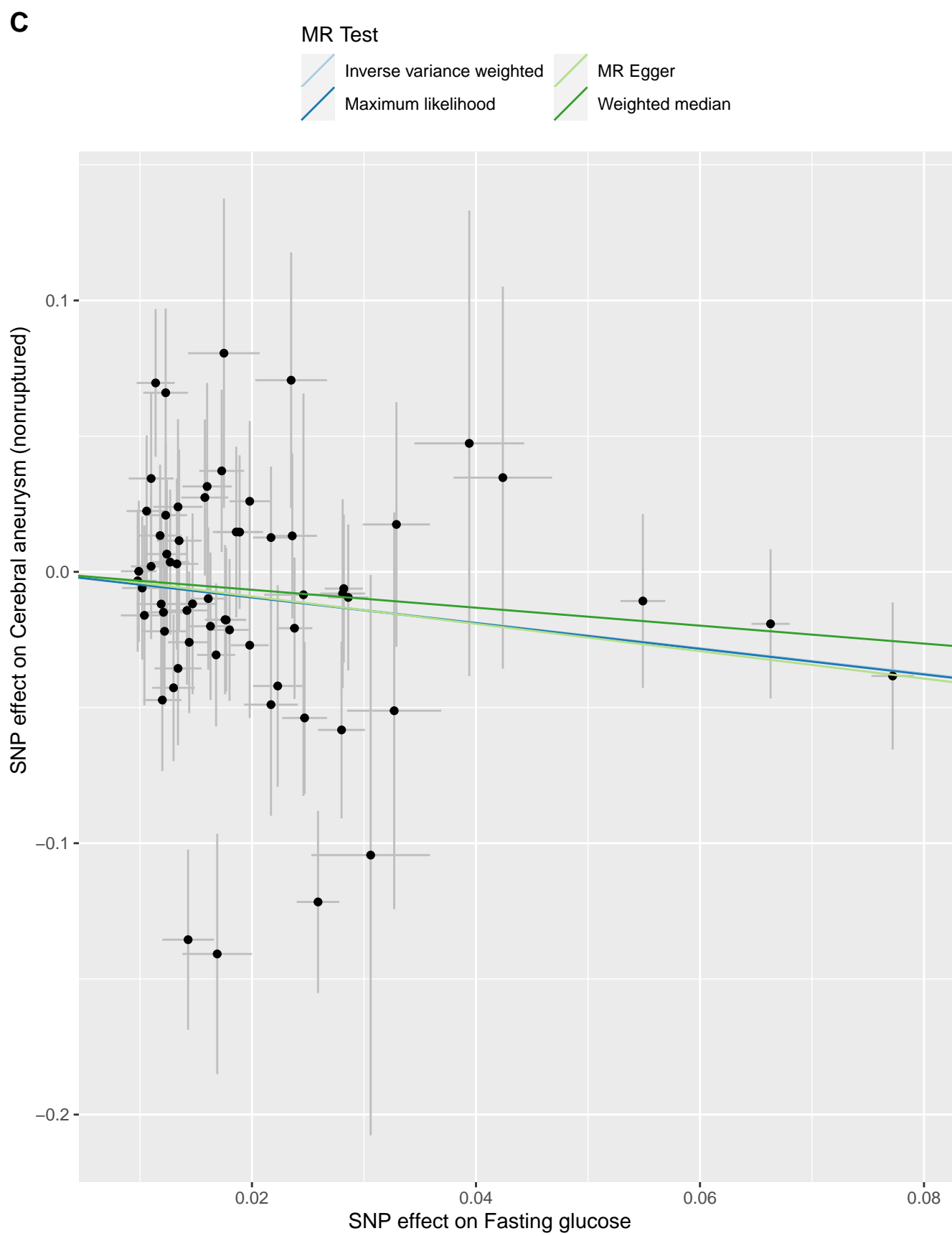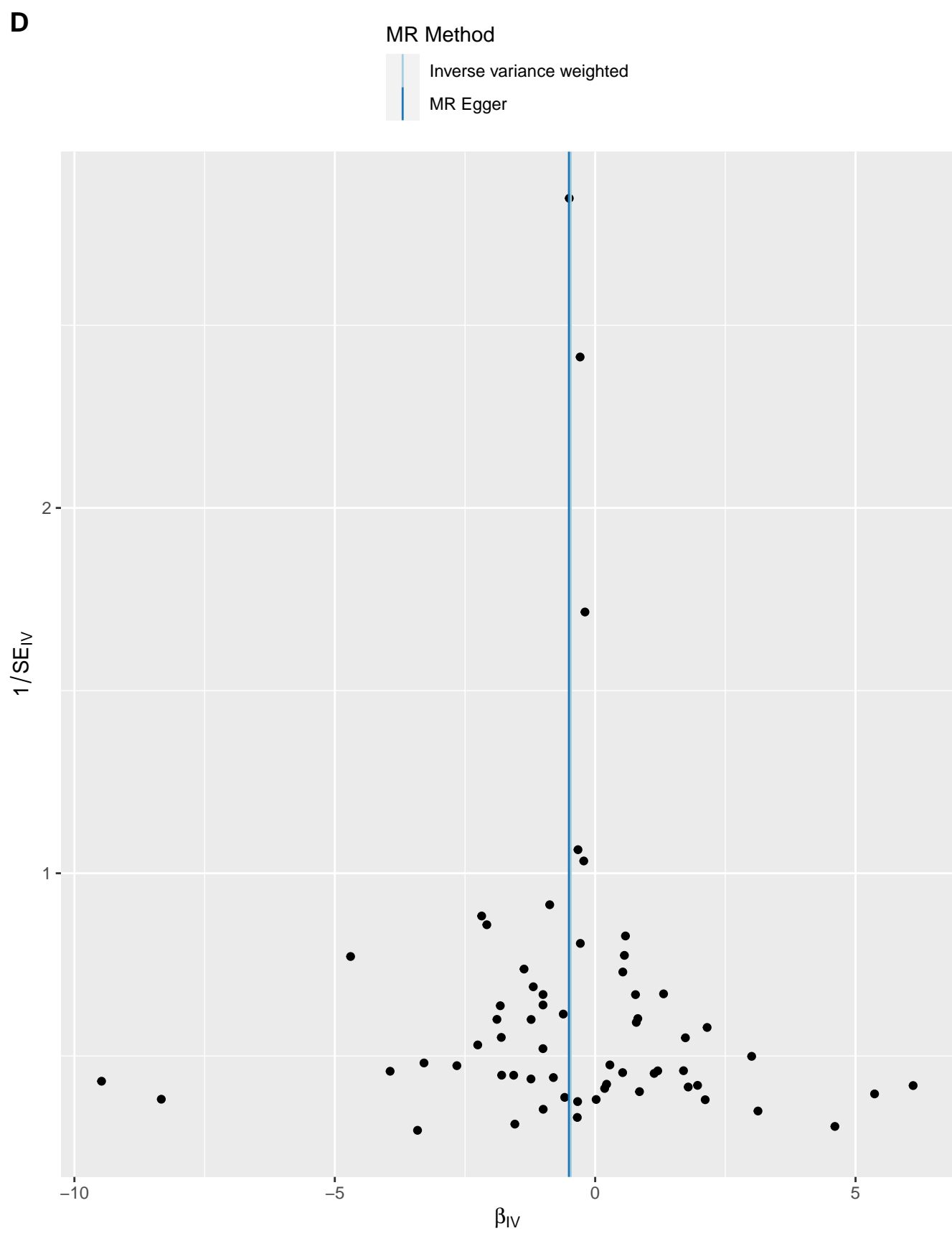

Supplement: Supplementary file 3 — Supporting Information [file BRB3-15-e70396-s002.pdf]

**A**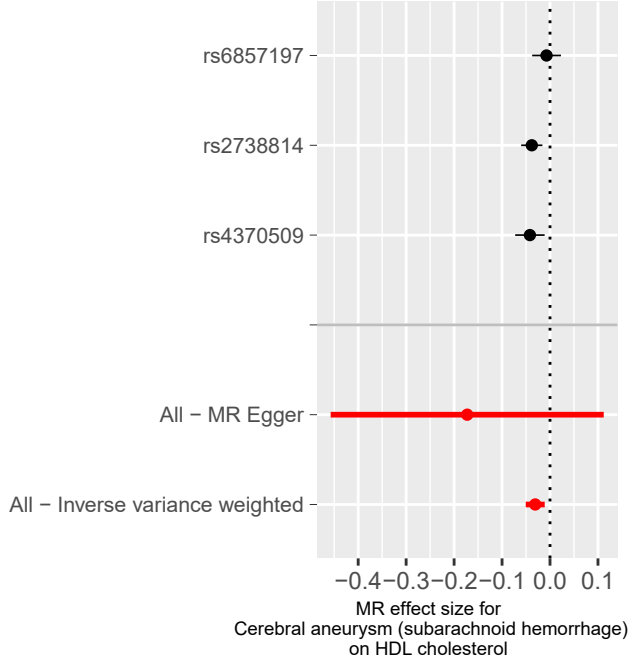**B**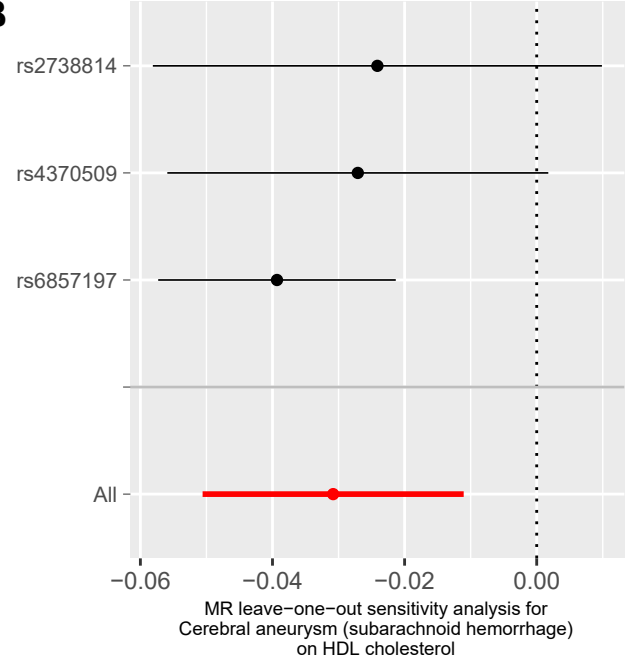**C**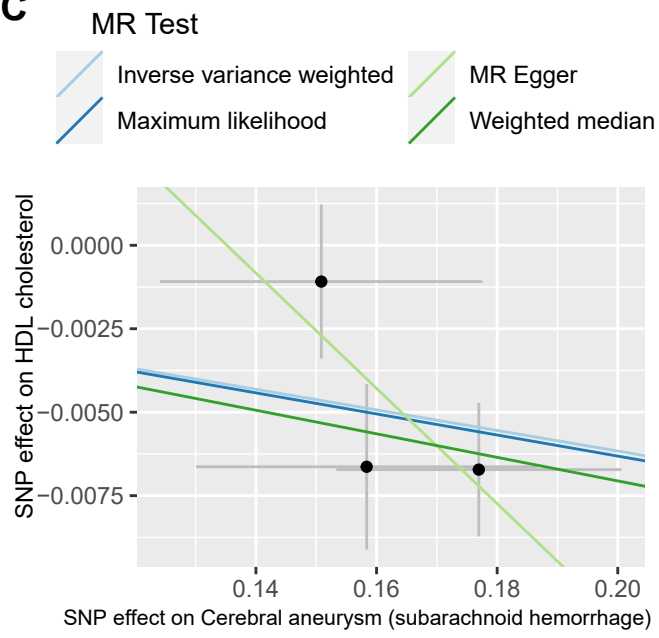**D**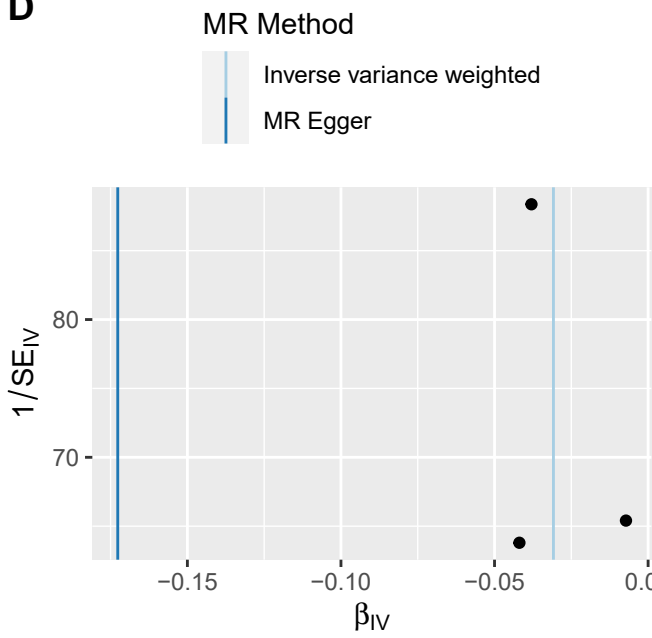

Supplement: Supplementary file 4 — Supporting Information [file BRB3-15-e70396-s005.pdf]

**A**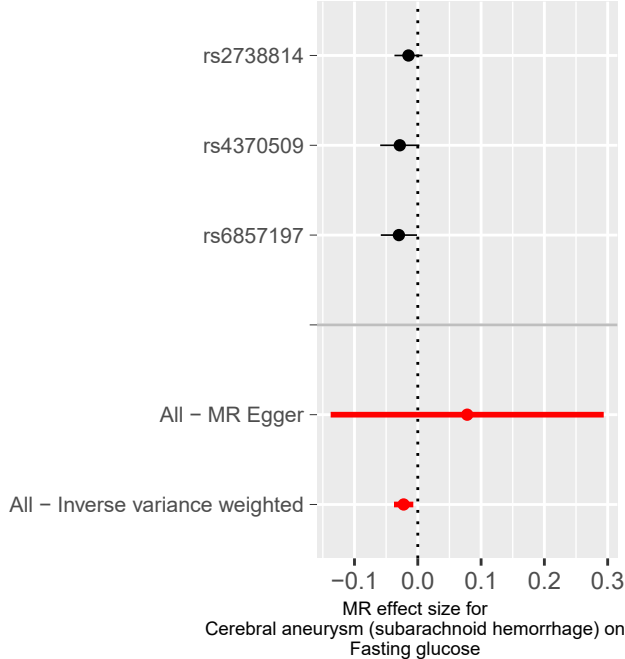**B**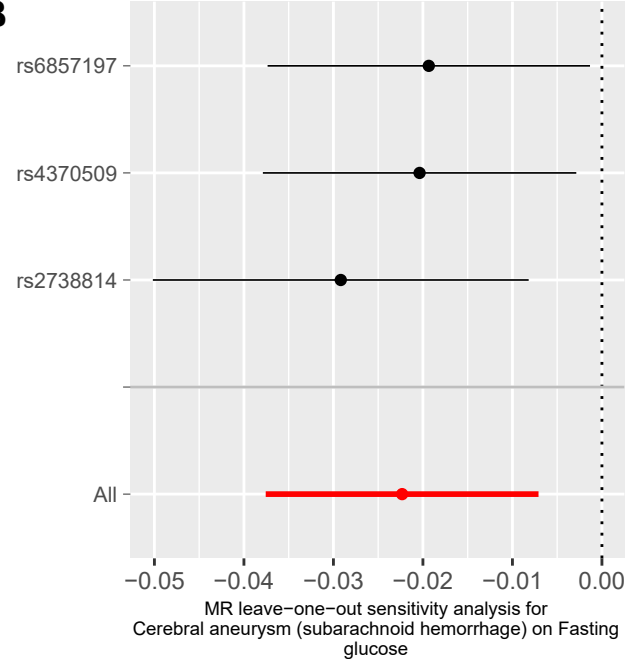**C**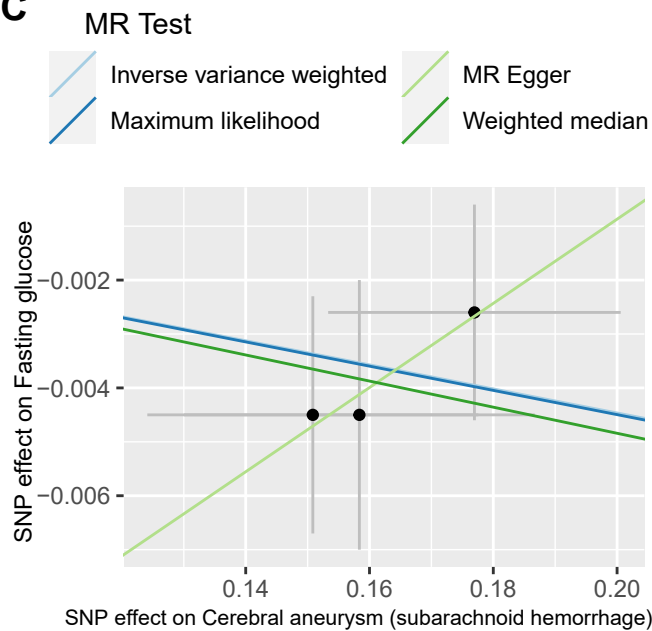**D**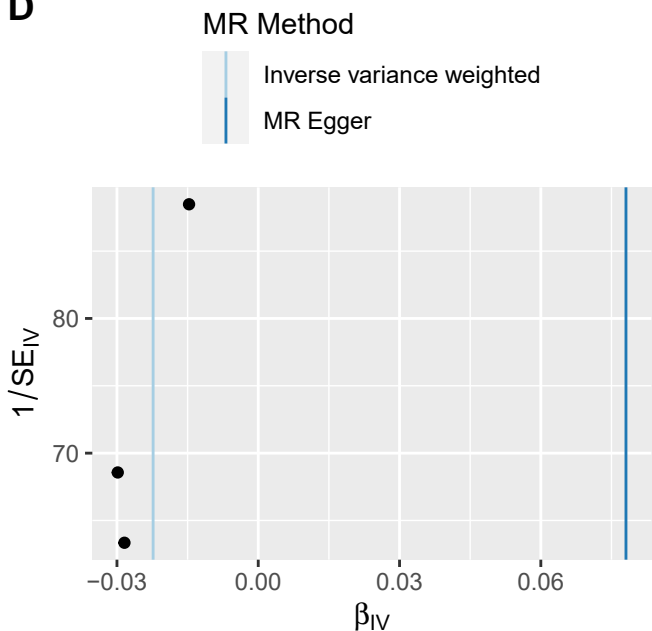

Supplement: Supplementary file 5 — Supporting Information [file BRB3-15-e70396-s004.pdf]
